# Versatility of cell-penetrating peptides for intracellular delivery of siRNA

**DOI:** 10.1080/10717544.2018.1543366

**Published:** 2018-12-31

**Authors:** Tejinder Singh, Akula S. N. Murthy, Hye-Jin Yang, Jungkyun Im

**Affiliations:** Department of Chemical Engineering, Soonchunhyang University, Asan, Republic of Korea

**Keywords:** Cell-penetrating peptides, conjugation, gene therapy, cellular internalization, siRNA delivery

## Abstract

The plasma membrane is a large barrier to systemic drug delivery into cells, and it limits the efficacy of drug cargo. This issue has been overcome using cell-penetrating peptides (CPPs). CPPs are short peptides (6–30 amino acid residues) that are potentially capable of intracellular penetration to deliver drug molecules. CPPs broadened biomedical applications and provide a means to deliver a range of biologically active molecules, such as small molecules, proteins, imaging agents, and pharmaceutical nanocarriers, across the plasma membrane with high efficacy and low toxicity. This review is focused on the versatility of CPPs and advanced approaches for siRNA delivery.

## Introduction

The internal cytosolic region is separated from the extracellular region by the plasma membrane, which plays a key role in maintaining osmotic balance, cellular homeostasis, and cellular uptake activities (Takeuchi & Futaki, 2016). The cytoplasmic membrane acts as a hydrophobic, defensive barrier that hampers the influx of many drugs, nucleic acids, peptides, and proteins into cells (Sawant & Torchilin, 2010). Only a few molecules possessing natural penetrating character and appropriate size, charge, and polarity are capable of directly passing through the membrane into the cytoplasm (Sawant & Torchilin, 2010). The cell membrane limits the permeability as well as the efficacy of macromolecules (e.g., nucleic acids) with high molecular weights (Margus et al., 2016).

Small interfering RNAs (siRNAs) lead to downregulation of target mRNAs and thus provide a promising pharmaceutical target in gene therapy (Vaissière et al., 2017; Xiang et al., 2017). However, due to their high molecular weight and the negative charge of the phosphate backbone, they cannot readily cross the cell membrane. Therefore, the clinical and therapeutic value of siRNAs is currently limited. Continuous research has been pursued to deliver siRNAs through the cell membrane into the cytoplasm, and various strategies have been developed to overcome this permeability issue (Nakase et al., 2013). Among them, viral and non-viral approaches have been the primary delivery strategies. Viral vectors are primitive vectors that have been utilized for gene therapy. These vectors have unique features that allow delivery of genetic material across the cell membrane with high efficacy. However, their success has been limited due to several challenges, for instance, limited cargo-carrying capacity, low delivery efficiency, the risk of mutation, high cytotoxicity, and lack of target specificity. In addition, viral vectors are not compatible with all kinds of nucleic acid-based molecules (e.g., short synthetic oligonucleotides) (Lehto et al., 2016). However, opportunities still exist to improve ways the process of viral vectorization (Lehto et al. 2012; Nakase et al. 2012).

Instead, a novel non-viral approach has been proposed to deliver a variety of macromolecules through the cell membrane, using positively charged amino acid residues that can penetrate the cell membrane. These amino acid residues are identified as non-homologous short peptides (LeCher et al., 2017) and have been alternatively referred to as protein transduction domains (PTDs), Trojan peptides, model amphipathic peptides (MAPs), membrane translocating sequences (MTSs), and, most frequently, cell-penetrating peptides (Langel, 2006; Madani et al., 2011).

The majority of CPPs consists mainly of arginine and lysine residues, making them cationic and hydrophilic. Occasionally, CPPs can be amphiphilic, anionic, or hydrophobic in nature (LeCher et al., 2017). CPPs are composed of 6–30 amino acids and, due to the short sequence length, they could be easily synthesized (Vive`s et al., 1997). Studies have revealed that the positive charge and amphipathic nature of CPPs are the critical features for cellular internalization and allow CPPs to carry macromolecules, polypeptides, and oligonucleotides across the cell membrane. Some CPPs are derived from natural biomolecules (for instance, Tat, an HIV-1 protein), while others (for instance, polyarginine) are obtained by synthetic methods. CPPs can have different origins and sequences and, thus, can exhibit various physicochemical properties (Guidotti et al., 2017) (Table 1).

**Table 1.** Examples of various CPP types.

CPP name	Sequence	Origin	Class	References
HIV-1 TAT protein, TAT_48-60_	GRKKRRQRRRPPQ	Natural (HIV-1 TAT protein)	Cationic	(Joliot et al., 1991)
Penetratin, pAntp_43-58_	RQIKIWFQNRRMKWKK	Natural (Antennapedia *Drosophilamelanogaster*)	Cationic	(Joliot et al., 1991; Derossi et al., 1994)
Polyarginines	R_n_	Synthetic	Cationic	(Futaki et al., 2001)
HRSV	RRIPNRRPRR	Natural	Cationic	(Milletti, 2012)
AIP6	RLRWR	natural	Cationic	(Milletti, 2012)
MPG	GALFLGFLGAAGSTMGAWSQPKKKRKV	HIV glycoprotein 41/ SV40 T antigen NLS	Membranotropic CPP/Primary Amphipathic	(Morris et al., 2008)
Pep-1	KETWWETWWTEWSQPKKRKV	’’	Membranotropic CPP/Primary Amphipathic	(Heitz et al., 2009)
ARF(1-22)	MVRRFLVTLRIRRACGPPRVRV	Natural	Membranotropic CPP/Primary Amphipathic	(Milleti, 2012)
pVEC	LLIILRRRIRKQAHAHSK	Natural	Membranotropic CPP/Primary Amphipathic	(Elmquist et al., 2001)
Transportan	GWTLNSAGYLLGKINLKALAALAKKIL	Synthetic	Membranotropic CPP/Primary Amphipathic	(Pooga et al., 1998)
MAP17	QLALQLALQALQAALQLA	Synthetic	Secondary amphipathic α-helical	(Milletti, 2012)
				
VT5	DPKGDPKGVTVTVTVTVTGKGDPKPD	Synthetic	Amphipathic (β-sheet)	(Oehlke et al., 1997)
Bac7	RRIRPRPPRLPRPRPRPLPFPRPG	Synthetic	Amphipathic &AMPs (Proline rich)	(Sadler et al., 2002)
(PPR)n	(PPR)3, (PPR)4, (PPR)5, (PPR)6	Synthetic	Proline-rich amphipathic CPPs	(Milletti, 2012)
gH625	NH_2_-HGLASTLTRWAHYNALIRAF-CONH_2_	Natural (Glycoprotein gH of HSV type I)	Membranotropic CPPs	(Smaldone et al., 2013)
GALA	WEAALAEALAEALAEHLAEALAEALEALAA	Synthetic	Membranotropic CPP /Secondary amphipathic α-helical	(Milletti, 2012)
INF7	GLFEAIEGFIENGWEGMIDGWYGC	Influenza HA2 fusion peptide	Membranotropic CPP /Secondary amphipathic α-helical	(El-Sayed et al., 2009)
CADY	GLWRALWRLLRSLWRLLWRA	PPTG1 peptide	Membranotropic CPP /Secondary amphipathic α-helical	(Konate et al., 2010)
C105Y	CSIPPEVKFNKPFVYLI	Natural(1-Antitrypsin)	Hydrophobic	(Rhee & Davis, 2006)
PFVYLI	PFVYLI	Synthetic	Hydrophobic	(Rhee & Davis, 2006)
Pep-7	SDLWEMMMVSLACQY	CHL8 peptide phage clone	Hydrophobic	(Gao et al., 2002)

**Table 2.** Examples of CPP-siRNA delivery.

CPP	Interactions	Proposed mechanism	Target gene	Results	References
MPG&MPGΔ^NLS^	Non-covalent	Non-endocytotic	Luciferase	Effective up to 80%-95%	(Simeoni et al., 2003)
Tat-LK15	Non-covalent	Endocytic	nNOS	Improving the stability of siRNA in serum, and downregulating the expression of nNOS effectively and specifically	(Peng et al., 2017)
pH responsive ACPP	Non-covalent	Endocytic	PLK-1	Selectivity towards tumor cells	(Xiang et al., 2017)
RICK	Non-covalent	Non-endocytotic	Luciferase & CyclinB1	Knockdown of the expression of luciferase ∼75% and 80% of endogenous CyclinB1	(Vaissière et al., 2017)
LMWP	Covalent	Non-endocytotic	EGFP	Improving siRNA delivery with high gene-silencing efficacy and low cytotoxicity	(Ye et al., 2017; Ye et al., 2018).
Modified Octa-arginine	Non-covalent	Endocytic	Survivin	Reduction of the gene expression up to ∼60%, and strong siRNA binding and delivery efficiency	(Li et al., 2015)
PF & NF (analogs of Transportan 10)	Non-covalent	Endocytic	Luciferase	Effective up to 65%-85%	(Veiman et al., 2013)
SPACE	Non-covalent	Endocytic	IL-10& GAPDH	Improving penetration of siRNA into skin, and enhanced internalization into epidermal keratinocytes	(Hsu & Mitragotri, 2011)
SHRss	Non-covalent	Endocytic	Luciferase	High efficacy, and low toxicity	(Tai et al., 2015)
gH625	Non-covalent	Non-endocytic	GFP	High efficacy, and limited cell toxicity	(Ben Djemaa et al., 2018)
RVG9R3LC	Non-covalent	Endocytic	Murine superoxide dismutase-1	Enhanced endosomal escape, high efficacy, and no significant cell toxicity	(Ullah et al., 2018)
BR2	Non-covalent	Endocytic	GFP	High transfection efficiency, specificity towards target cells, and no significant cytotoxicity	(Lee et al., 2018)
LKH-stEK	Non-covalent	Endocytic	CTGF	High gene knockdown efficiency, and enhanced endosomal escape	(Hyun et al., 2018)

Originating from the trans-activator of transcription (Tat) protein of human immunodeficiency virus type 1 (HIV-1), the Tat peptide was the first peptide identified as a CPP. In this protein, the translocation domain, which is responsible for cellular internalization, was found to be a sequence of 11 amino acids (YGRKKRRQRRR) (Vivès et al., 1997). MPG (N-Methylpurine DNA Glycosylase) is another example of a CPP, synthesized from the fusion of the HIV glycoprotein 41 sequence (hydrophobic domain) and the nuclear localization sequences of the SV40 T-antigen (hydrophilic domain). It exhibits a strong affinity toward single- and double-stranded oligonucleotides and is capable of cellular internalization (Guidotti et al., 2017). Here, we provide a broad overview of CPP classification, modes of cellular uptake, insights of CPPs, and introduce advanced approaches to CPP-mediated siRNA delivery.

## CPP classification

On the basis of amino acid sequence, origin, function, and pathways of cellular uptake, several methods have been proposed to classify CPPs; however, there are currently no criteria for CPP classification. Nevertheless, CPPs can be divided into four primary classes based on their physical and chemical properties: Cationic CPPs; Amphipathic CPPs; Membranotropic CPPs (Falanga et al., 2015; Galdiero et al., 2015); Hydrophobic CPPs (Jafari et al., 2015; Guo et al., 2016).

### Cationic CPPs

Cationic CPPs have a highly positive charge derived from their amino acid residues, for example, arginine and lysine. They include the Tat peptide, penetratin, and polyarginine (Wender et al., 2000; Guidotti et al., 2017). Studies of cationic CPPs have shown that the positive charge and the number of amino acids in the peptide sequence are directly responsible for membrane translocation. Arginine contains a guanidinium head group, which further contains positive charges that participate in hydrogen bond formation and electrostatic interactions with the negatively charged functional groups of the cell membrane. As a result, arginine confers more efficient internalization capability than lysine residues (Park et al., 2002). It was shown that at less than six arginine residues, the peptide lost its function to translocate itself into the cytosol. On the other hand, increasing the number of arginine residues to more than six greatly enhanced the translocation efficiency of the CPP complexes (Wender et al., 2000; Guidotti et al., 2017).

Some cationic CPPs can be referred to as antimicrobial peptides (AMPs) since they have the ability to cross the host cell membrane as well as to kill the bacterial cells (Joanne et al., 2009). For example, the fusion domain of influenza virus that helps entering the host cell was evaluated for its antibacterial activity and the study has shown that the amidation of the C-terminus of the domain can improve the efficacy (Ye, 2018). Sometimes, there is a drawback that a high concentration of cationic CPPs can disturb the cell membrane, which can lead to cytotoxicity, apoptosis, and hemolytic activity (Saar et al., 2005).

### Amphipathic CPPs

Amphipathic CPPs are chimeric or fused peptides, constructed from different sources, and contain both positively and negatively charged amino acid sequences (Guidotti et al., 2017). Amphipathic CPPs are prepared by fusing hydrophilic and hydrophobic domains. For example, the representative amphipathic CPP, transportan is a chimeric CPP made up of the neuropeptide galanin and the mastoparan peptide, which is a peptide toxin from wasp venom (Avci et al., 2018; Fanghänel et al., 2014). It has the ability to transfect large hydrophilic drug molecules into the cell without membrane disruption (Pooga et al., 1998; Sawant & Torchilin, 2010). Amphipathic peptides can be subdivided into primary amphipathic, secondary amphipathic α-helical, β-sheet amphipathic, and proline-rich CPPs (Zaro & Shen, 2015; Guidotti et al., 2017).

Many primary amphipathic CPPs are obtained through a covalent bond between a cationic sequence and a hydrophobic sequence that is used to target the cell membrane (Ragin et al., 2002; Guo et al., 2016). For example, a short cationic peptide in the primary amphipathic CPP, such as a nuclear localization signal (NLS), can be utilized to deliver cargo into the nucleus via a nuclear pore formation process. Amphipathic α-helical peptides contain a highly hydrophobic domain on one side and a cationic, anionic, or polar domain on the other side. β-sheet amphipathic peptides are composed of one hydrophobic stretch and one hydrophilic stretch of amino acids, both of which are on opposite sides (Milletti, 2012). VT5 is an example of a β-sheet amphipathic peptide and has the ability to form β-sheets for cellular internalization (Guo et al., 2016). Proline-rich CPPs are another type of amphipathic peptides that are efficient in cellular uptake and have low cytotoxicity (Pujals & Giralt, 2008). They include bactenecin-7 (Bac7) (Sadler et al., 2002), proline-rich peptide (PPR)_n_ (Daniels & Schepartz, 2007), and SAP (VRLPPP)_3_, etc.

### Membranotropic CPPs

The membranotropic CPPs usually fall under the amphipathic class because they also exhibit both hydrophobic and amphipathic nature simultaneously. Their specific physical properties are featured by the presence of both large aromatic residues and small residues. Membranotropic CPPs contain a high content of alanine, glycine residues and also prolines to some extent, which confer the intrinsic conformational flexibility crucial for interacting to the cell membrane. Additionally, the existence of aromatic residues in the membranotropic CPPs is distinctive feature different from other hydrophobic CPPs. These hydrophobic residues play an important role in the favorable interactions between the CPP and the cell membrane (Falanga et al., 2015; Galdiero et al., 2015). The internalization process of membranotropic peptides will be discussed further in the mechanism section.

The membranotropic peptide can be derived from a viral fusion peptide, and gH625 is a well-studied viral peptide which is a part of glycoprotein H of Herpes simplex virus type I. gH625 is a membrane-perturbing domain enriched with hydrophobic residues, and aromatic residues such as tryptophan and tyrosine. The hydrophobicity and amphipathicity are the key features of gH625 that make the peptide interact with membrane lipids and form a transient helical structure to induce membrane organization temporarily. Thereby, it was demonstrated that gH625 is able to cross the cell membrane and to transport many conjugated cargoes into the cytosol (Falanga, Lombardi, et al., 2017; Falanga, Valiante, et al., 2017; Falanga, Galdiero et al., 2018).

E. Perillo et al. reported the synthesis and in vitro evaluation of multifunctional nanoparticles (NPs) composed of a superparamagnetic iron oxide nanoparticle (SPION) core, a cyanine fluorescent dye, polyethylene glycol polymer (PEG_5000_) and the membranotropic peptide gH625. Both CPP-capped and non-capped nanosystems were prepared and the results showed that the SPIONs-PEG-CPP NPs cell uptake was 3-fold higher than that for the NPs without CPP (Perillo et al., 2017; Ben Djemaa et al., 2018). Recently, it was also reported that gH625 is permeable to the blood-brain barrier (BBB) and can enter the rat brain *in vivo* without toxic effects. gH625 showed high tissue distribution toward the brain despite the liver withdrawal of the peptide (Falanga, Iachetta, et al., 2018; Tudisco et al., 2018).

### Hydrophobic CPPs

Hydrophobic CPPs contain only non-polar motifs or residues, which have relatively low net charge. Very few hydrophobic CPPs have been discovered and show differing behaviors compared to other kinds of CPPs (Marks et al., 2011; Guidotti et al., 2017). Based on modification type and natural amino acid sequences, hydrophobic CPPs can be further divided into four sub-classes: linear hydrophobic peptides, stapled peptides, prenylated peptides, and pepducins (Milletti, 2012; Guo et al., 2016). SG3, PFVYLI, pep-7, and fibroblast growth factors (FGF) are examples of hydrophobic CPPs (Guo et al., 2016; Guidotti et al., 2017).

## Mechanisms of cellular internalization

CPP internalization mechanism studies have been the most actively studied area since the discovery of the Tat peptide (Mueller et al., 2008; Choi & David, 2014). There is no single, prevailing cellular uptake pathway, and it was found that the mode of cellular uptake can be variable between experimental settings. In addition, reports have revealed that cellular uptake pathways can vary according to the nature of the CPP, cell lines, and attached drug or cargo (Stewart et al., 2008; Hirose et al., 2012). Thus, a CPP can use more than one route to enter the cell depending on the surroundings (Pujals et al., 2006; Fretz et al., 2007; Kosuge et al., 2008; Stewart et al., 2008; Madani et al., 2011; Cardoso et al., 2012; Hirose et al., 2012; Koren & Torchilin, 2012; Palm-Apergi et al., 2012; Mayor et al., 2014). Although it has been widely accepted that most CPPs enter the cell via endocytosis (Hirose et al., 2012; Palm-Apergi et al., 2012), it is assumed that typically three CPP cellular entry types affect the intracellular transport. They are direct or energy-independent internalization, energy-dependent endocytosis, and internalization through the formation of a temporary membrane structure (Fretz et al., 2007; Kosuge et al., 2008; Madani et al., 2011).

### Direct or energy-independent internalization

In most cases, internalization begins with the electrostatic interaction between the positively charged CPP and the negatively charged phospholipids of the cell membrane. Proteoglycans are a heterogeneous group of protein-carbohydrate complexes containing long, linear chains of glycosaminoglycan (GAGs) polysaccharides. The negatively charged carboxylate, phosphate, and sulfate groups of proteoglycans function as a receptor for extracellular CPPs.

After this initial association, subsequent internalization processes are described by several models. First, the ‘carpet-like’ mechanism, also known as the membrane-thinning model, occurs when the concentration of the penetrating peptides is above a threshold level. Through electrostatic interactions between CPPs and the cell membrane, a carpet-like structure is formed on the outer leaflet of the membrane, which spans the cell membrane surface until transient pores are formed. (Madani et al., 2011). Eventually, during this process, unfavorable peptide-membrane interactions disintegrate the membrane, making the cell membrane permeable with forming holes. Second, according to the ‘pore formation’ model, CPPs can enter through the plasma membrane either by toroidal pores or barrel-stave pores. In regard to toroidal pores, CPPs are inserted perpendicularly into the cell membrane without peptide-peptide interactions, leading to a pore formation. In the barrel-stave model, amphipathic α-helical CPPs create barrel-like structures in which the hydrophobic surface of the CPP can interact with the lipid core of the cellular membrane, while the hydrophilic part of the CPP points inward of the pore to produce a hydrophilic cavity that facilitates CPP entry into cells. In both mechanisms, a high concentration of the CPP is essential. Cationic CPPs and AMPs generally have the tendency to follow these pore-dependent mechanisms (Guidotti et al., 2017; Kumar et al., 2018).

**Figure 1.**
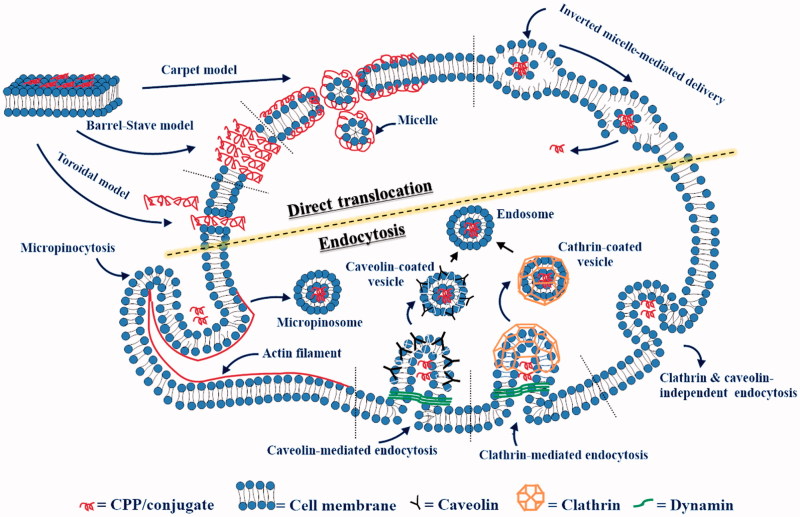
Diagram of cellular uptake mechanisms.

Conversely, especially in the case of amphipathic and membranotropic peptides, the initial interaction begins with the association between the hydrophobic sequences and the membrane bilayer. Thereby the peptide can penetrate into the membrane core and associates with themselves at the lipid/water interface. This leads to local and temporary perturbation of the cell membrane and the direct translocation of the CPP becomes possible. Besides, the translocation of membranotropic peptides can be also described by an inverted micelle model (Falanga et al., 2015). The hydrophobic residues of the CPP interact with the lipids of the cell membrane to form micelles. These micelles internalize and release the peptide into the cytosol through inversion of the micelles (Figure 1). This uptake pathway is not feasible with highly cationic CPPs, such as polyarginine and Tat (Madani et al., 2011; Guidotti et al., 2017).

### Endocytosis

Endocytosis is a natural and energy-dependent process in which cells gain energy from the extracellular region during the translocation. (Guo et al., 2016). This mechanism primarily consists of two pathways: phagocytosis, which engulfs larger substances such as viruses, bacteria, or other particles, and pinocytosis, which is utilized for taking in fluids and dissolved solutes. Pinocytosis occurs in all cell lines and can be further sub-divided into clathrin-mediated, caveolin-mediated, clathrin- and caveolin-independent endocytosis, and macropinocytosis (Koren & Torchilin, 2012; Mayor et al., 2014).

The most understood pathway is the clathrin-mediated endocytosis which is constitutive to all mammalian cells. It is initiated and activated by binding of molecules to their corresponding transmembrane receptors and then the bound ligand-receptor complexes begin to form coated-pits mainly by clathrin, a representative coat-protein for vesicle formation(Sawant & Torchilin, 2010). The coated pits are invaginated with the support of actin, intersectin, cortactin, and epsin proteins. Subsequently, the GTPase dynamin is recruited to the neck of the coated pit which leads to the membrane fission. The clathrin-coated vesicles finally become endocytic. (Schafer, 2002; Sauvonnet et al., 2005; Messa et al., 2014). The caveolin-mediated pathway is a receptor-independent pathway that exhibits some similarities to the clathrin-mediated pathway because it also forms a flask-shaped pit, so-called caveolae (LeCher et al., 2017). The main protein involved in this process is caveolin which is an integral membrane protein that forms tight interactions with cholesterol present in the cell membrane. Thus, caveolin coat could be invaginated and pinched off from the cell membrane particularly in the cholesterol and sphingolipid-rich microdomains of the cell membrane (Roy & Wrana, 2005). This pathway is often referred to as detergent-resistant membranes (DRMs), detergent-insoluble glycolipid-enriched complexes (DIGs), or glycosphingolipid enriched membranes (GEMs) (Triantafilou et al., 2002). Macropinocytosis occurs constitutively in many cells to form a rather large vacuoles known as macropinosomes. In response to the cellular signaling, the process is initiated by an actin-mediated disruption of the plasma membrane (Lim & Gleeson, 2011). Ruffling and invagination of the cell membrane occurs and the resulting macropinosome will take up molecules from the extracellular fluid and finally deliver them into the cells (Bolhassani et al., 2017). Lastly, clathrin- and caveolae-independent endocytosis is distinct from the other endocytic pathways in that dynamin is not involved in the process. It has been proposed interleukin-2 receptor was internalized by generating a raft from the cell membrane. However, this process has not been studied much yet and is poorly understood (Figure 1).

During the internalization process, CPPs and CPP-cargo complexes often remain trapped inside the endosomes. This is a major limiting factor for efficient internalization (Berg & Dowdy, 2011). CPPs and cargo must escape the endosomes to avoid degradation by diverse enzymes in lysosomes (Vasconcelos et al., 2013). Many agents have been developed to improve endosomal release of CPPs and CPP-associated complexes. For instance, histidine moieties (C-5H-Tat-5H-C) in CPPs absorb protons from the endosomes, leading to an increase in endosomal pH and osmotic pressure. The increased osmotic pressure breaks the membrane of the endosomal vesicle and facilitates the release of the endosomal contents (Sugita et al., 2007). Similarly, a pH-sensitive fusogenic dTAT-HA2 CPP is capable of destabilizing endosomal membranes and can facilitate CPP release from endocytic vesicles. (Lundberg et al., 2007).

In fact, CPP-cell membrane interactions are quite complex and each mode of internalization is variable according to the CPP sequence, length, charge, structure, concentration, and the cell lines (Madani et al., 2011). In general, cationic CPPs are taken up by endocytosis at low CPP concentration, but also possible by direct translocation at high concentration (Tunnemann, 2006). However, due to the amphipathicity, membranotropic CPPs can follow direct internalization regardless of their concentration. It is advantageous that such CPPs can circumvent the endosomal entrapment either by escaping from the inverted micelle or by direct translocation. In contrast, cationic CPPs could be entrapped in the endosomes and degradation processes are unavoidable. In addition, the cargoes of the cationic CPPs could not reach the intracellular target. Therefore, membranotropic peptides can directly diffuse into the cytosol and provide immediate bioavailability and efficacy (Falanga et al., 2015). In addition, if a drug is attached to the membranotropic peptide, the cargo could be efficiently delivered by the pathways mentioned above. This kind of modifying the internalization mechanism of a drug can modulate the toxicity of the drug and may help to overcome the drug resistance problems.

### Translocation through the formation of a transitory membrane structure

Translocation through the formation of a transitory membrane structure is based on the formation of stable inverted micelles in the middle region of the cell membrane. (Bolhassani et al., 2017). This process is initiated when the attractive potential between the cationic CPPs, particularly arginine-enriched CPPs, and the lipid heads of the membrane is strong. As a result, the CPP is transported into the cell membrane to form a temporary inverted micelle. In such an environment, the CPP is stabilized by decreasing the potential energy of the CPP. Finally, the CPP is delivered into the cell without forming a vesicle.

## CPP and drug molecule complex formation

In general, conjugation of CPPs and drug molecules can be either through covalent bonding or non-covalent complex formation via charge interactions (Kawamoto et al., 2011). Initially, covalent conjugation was the most common method for producing a well-defined biologically active molecule. This strategy creates a strong bond between the CPP and cargo molecule via a chemical linkage; for instance, a disulfide or thio-ester bond (Guidotti et al., 2017). Among the chemical linkages, a disulfide linkage has been used most often, and the bond can be easily cleaved by cytoplasmic degradation enzymes. However, covalent conjugation methods have drawbacks. Strong bonding between the CPP and drug molecule can potentially disrupt the biological characteristics of the drug molecule (Guidotti et al., 2017). This method of conjugation is not as compatible with the delivery of large molecular weight cargo, such as pDNA and oligonucleotides. In addition, conjugation requires multiple steps of synthesis and purification that can be time-consuming and laborious (Lehto et al., 2012).

The nanocomplex formation can also be conducted via non-covalent interaction. This method is primarily based on electrostatic interactions and hydrophilic-hydrophobic interactions between the CPPs and cargo molecules (Regberg et al., 2012). Amphipathic peptides such as MPG and penetratin can form stable non-covalent complexes with siRNA at certain molar and charge ratios (Deshayes et al., 2008; Layek et al., 2015). The first study on non-covalent interactions was reported by E. Gros et al. (2006) utilizing MPG CPP for the delivery of short nucleic acids, pDNA, peptides, proteins, and siRNA . This study had shown that there is a strong affinity of CPP for charged molecules, even at a nanoscale concentration (Layek et al., 2015). The main advantages of this strategy are the relatively simple preparation and the retention of drug cargo properties (Huang et al., 2015). In contrast to covalent conjugation, this approach has been used less frequently due to the inability of CPPs to condense some cargo into stable nanocomplexes when the molar and charge ratios are inappropriate (Foged & Nielsen, 2008; Lehto et al., 2012).

## Factors for internalization

Intracellular delivery of a CPP-cargo complex is dependent on the size of the complex itself and affects the pharmacokinetics and pharmacodynamics of the drug molecules. The size of a nanocomplex consisting of CPP and siRNA needs to be less than 200 nm in order to ensure cellular uptake and tissue distribution and to overcome the risk of cellular toxicity (Reischl & Zimmer, 2009; Maeda, 2010; Wang et al., 2010; Gooding et al., 2012; Van Asbeck et al., 2013; Xu & Wang, 2015; Aldrian et al., 2017).

**Figure 2.**
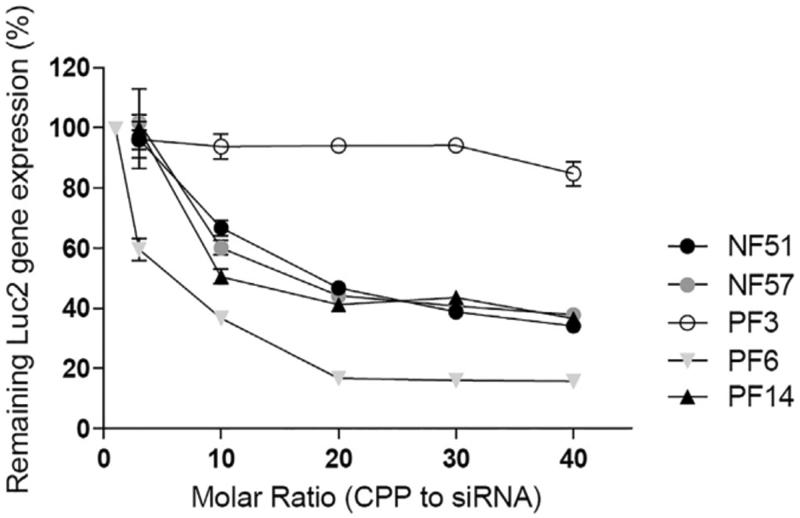
Downregulation of luciferase (Luc2) gene (Pärnaste et al., 2017). Figure reproduced from the respective publisher.

The molar ratio is defined as the number of CPPs required per siRNA molecule. It is an important factor that influences the stability of CPP-siRNA complexes. L. Pärnaste et al. (2017) had examined the effect of molar ratio on five different CPP types, which were modified derivatives of the transportan10 peptide. One of them, PF6, was coupled with siRNAs at a molar ratio of 1:20, which showed the highest transfection efficiency among the CPP-siRNA complexes (Figure 2). This complex was more effective (up to 80%) at down-regulating luciferase expression than the other CPP-siRNA complexes. In all cases, molar ratios higher than 1:15 had shown improved results in down-regulating luciferase expression. From these results, it was suggested that a higher CPP concentration is needed to fully condense the siRNA, and can consequently lead to the formation of stable nanoparticles for successful delivery.

The CPP net charge and environmental pH are important parameters for CPP-siRNA binding and complex stability. The charge ratio (CR) is defined as the number of positive charges (from the peptide) per negative charge (from the siRNA). CPP requirements for the optimal CR change with pH. A difference was observed with PF6 when the pH value was changed (Pärnaste et al., 2017). It had been determined that 15 CPPs per siRNA were needed at pH 7.4, while only 6 CPPs per siRNA were needed at pH 5.5 in order to form a stable complex.

**Figure 3.**
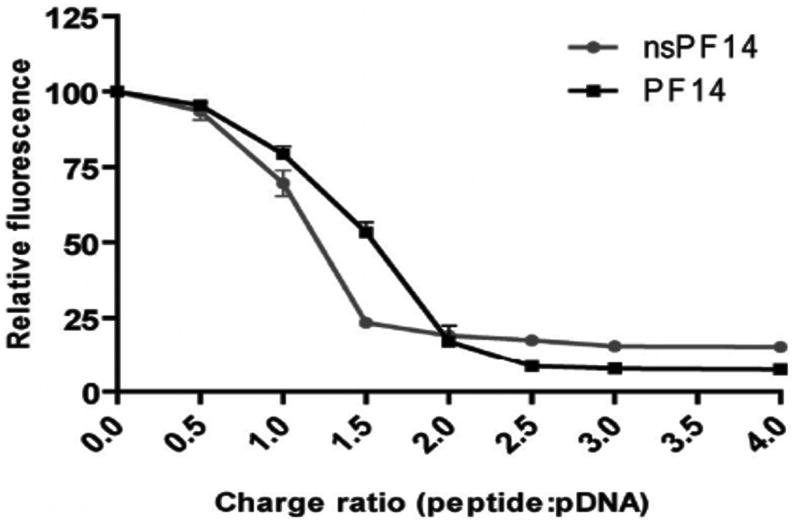
Comparison of the pDNA condensation efficiency by PF14 and nsPF14 (Veiman et al.,^2013^). Figure reproduced from the respective publisher.

Higher CPP concentrations can cause possible aggregation of CPP-cargo nanocomplexes via electrostatic interactions, which results in an increase in hydrodynamic diameter of nanocomplexes (Veiman et al., 2013). However, appropriate CR between CPP and cargo can resolve this issue (Margus et al., 2016). K. Veiman et al. (2013) had determined that CPP PF14 (an analog of the transportan peptide) can condense the pDNA at a CR of 2:1 into stable nanocomplexes (Figure 3). The authors had reported that, at CR2, the nanocomplex size was approximately 135 nm; whereas the size was different at other CRs. Y. Li et al., (2015) had reported that a CR of at least 4:1 is required for arginine derivatives to condense siRNA completely, demonstrating the point at which the best binding efficiency between arginine (R8) derivatives and siRNA was reached. These studies suggest that an optimal CR is essential for preparing stable complexes.

## CPP-mediated delivery of siRNA

Gene therapy and chemotherapy generally rely on suitable delivery systems that mostly lack the ability to only target specific cells. This issue can cause cytotoxic and/or adverse effects for both normal and cancerous cells. As an alternative, many antisense-based strategies are currently being developed; due to their specific nature, they are being intensely studied for gene expression regulation (Kole et al., 2012; Lehto et al., 2012).

In particular, siRNA is perceived to be a promising therapeutic with several benefits, including high gene specificity. Despite its potency in gene therapy, the utility of siRNA is limited because it is biologically unstable and has poor cell membrane permeability (Xiang et al., 2017). To address these issues, CPP-mediated delivery of siRNA has been used. Since it had been shown that CPPs can translocate macromolecules into cells both *in vitro* and *in vivo* (Bolhassani et al., 2017; Guidotti et al., 2017), CPPs have gained great attention as proficient drug carriers in the field of gene therapy. Some recent strategies will be discussed herein, which have shown high efficacy, high gene knockdown efficiency, and low cytotoxicity. F. Simeoni et al. (2003) first introduced the delivery of siRNA into mammalian cells with a CPP MPG. In the study, the CPPs formed stable non-covalent complexes with siRNAs, leading to down-regulation of gene expression. The authors determined that the parent MPG and a modified form of MPG (MPGΔ^NLS^) exhibited strong potential for siRNA translocation. Both CPP nanocomplexes were incubated at a molar ratio of 1:10 and applied to HeLa and Cos-7 cells. The results revealed that MPG-siRNA nanocomplexes suppressed luciferase activity up to 78% and 85% in HeLa and Cos-7 cells, respectively. In contrast, the modified MPGΔ^NLS^-siRNA nanocomplexes dramatically increased the effect to about 90% and 95% in HeLa and Cos-7 cells, respectively.

Recently, a series of studies has demonstrated that nitric oxide (NO) and neuronal nitric oxide synthase (nNOS) are the primary constituents in acute pain, including neuropathic and inflammatory pain. NO is known to be synthesized from arginine via nNOS activity in the nervous system. J. Peng et al. (2017) had reported on a modified CPP, Tat-LK15, which could form a non-covalent nanocomplex (Tat-LK15-siRNA) with siRNA for nNOS downregulation. The stability, efficiency, and selectivity of this nanocomplex were assessed in rat neuronal cells. Compared to the traditional siRNA carrier Lipofectamine^TM^, it was observed that this CPP can deliver nNOS-siRNA more effectively and specifically into rat cells. The CPP and siRNA nanocomplexes were formed at a ratio of 2:1 (w/w) and have shown good stability in serum for more than 8 h. Using MAP2 (Microtubule Associated Proteins 2) staining, it was observed that Tat-LK15-siRNA nanocomplexes were completely internalized into the cells. From these results, it could be inferred that Tat-LK15 can deliver siRNA proficiently to treat neuropathic diseases by regulating nNOS expression in cells and to improve the stability of siRNA in serum.

In another study, B. Xiang et al. (2017) had developed a pH-responsive ACPP (activatable cell-penetrating peptide) consisting of three units: CPP (octa-arginine), a polyanionic inhibitory sequence composed of histidine and glutamic acid residues, and an acid cleavable linker. The polyanionic domain shielded the cationic moieties of ACPP through intramolecular electrostatic interactions. The hydrazone bond in the acid cleavable linker breaks when ACPP is exposed to an acidic pH environment near tumor cells, leading to an unshielded, or activated, ACPP. The activated ACPP would potentially show enhanced intracellular uptake activity. Two types of CPP-siRNA complexes were prepared and tested. In the first complex, ACPP was conjugated with a liposomal carrier, DSPE-PEG2000-NHS (1:1.2 molar ratio). In the second complex, a non-modified CPP (octa-arginine) was conjugated with the same liposomal carrier. The authors reported that only ACPP-mediated nanocomplexes showed a significant response to the acidic pH, improved cellular uptake, down-regulated PLK-1 gene expression, and increased apoptosis in MCF-7 cells.

A. Vaissière et al. (2017) had developed a stable RICK (**R**etro-**I**nverso **C**ADY-**K**) CPP modified from a secondary amphipathic peptide. This CPP was designed using D-amino acids to generate inversion in the peptide sequence. Among the D-amino acids, tryptophan played a significant role in membrane translocation via interaction with GAGs (Bechara et al., 2013; Jobin et al., 2015). The unique feature of this CPP is that it maintained the biophysical properties of the nanocomplexes both in serum and serum-free medium. RICK-based nanocomplexes have shown the ability to internalize siRNA rapidly into cells. This is the key factor to overcome the risk of enzymatic degradation. These nanocomplexes exhibited remarkable gene knockdown efficiency even at a low dose and without any significant cell toxicity. For example, up to ∼75% luciferase and ∼80% endogenous CyclinB1 gene expression reductions were observed at 1:20 nM siRNA.

Recently, a LMWP (Low Molecular Weight Protamine) CPP was developed by J. Ye et al. (2017). In their work, a covalent conjugate, LMWP-siRNA, was synthesized via a disulfide linkage, which is easily cleavable in the cytosol for siRNA release. Moreover, a PEG (PolyEthylene Glycol) polymer chain was inserted between LMWP and the siRNA to prevent self-assembly through intermolecular interactions between the cationic CPP and the anionic siRNA. The authors insisted that the chemically conjugated complex showed better efficacy and EGFP gene silencing in vitro than the charge-coupled CPP-siRNA. The authors further investigated the route of internalization of LMWP-siRNA conjugates and in vitro cytotoxicity. It was demonstrated that the conjugates might be internalized into the cells via multiple pathways including direct internalization and clathrin- and caveolae-independent endocytic pathway. It was also indicated that these covalent conjugates do not exhibit significant cell toxicity and can be suitable candidates for siRNA delivery (Ye et al., 2018).

Since siRNA is known to be rapidly degraded by nucleases in serum, development of CPP-siRNA complexes requires great care and specific design strategies. Y. Li et al. (2015) have proposed four modified derivatives of octa-arginine (R8) CPP conjugated with three different types of fatty acids: stearic acid (StA), oleic acid (OA), or linoleic acid (LA). All of these modified CPP-siRNA nanocomplexes at 4:1 CR were incubated in culture media containing 10% or 25% FBS (fetal bovine serum) at 37 °C. In previous studies, the stability non-modified CPP-siRNA was reported to decrease rapidly as incubation time increased. However, when the modified R8 with siRNA at a CR of 4:1 were incubated in 25% FBS, the conjugates retained stability for up to 24 hr. Compared to the non-modified R8, modified R8 CPPs such as StA-R8, OA-R8, and LA-R8 with siRNA could show better efficiency for down-regulating gene expression (up to ∼60%). Furthermore, they exhibited stronger siRNA binding, higher stability, and more efficient translocation of the nanocomplexes across the cell membrane.

L. Pärnaste et al. (2017) had reported five modified derivatives of the transportan10 CPP and had examined the parameters for the formation of CPP-siRNA nanocomplexes, for the binding properties of the CPPs to the siRNAs, and for the stability of the condensed nanocomplexes. Out of the five tested CPPs, PF6, which is modified with the pH-sensitive moiety chloroquine, had shown the highest transfection efficiency at a molar ratio of 1:20. The pH of the surrounding environment is a major factor for condensing the siRNA into a stable nanocomplex and for protecting the siRNA from nuclease degradation. Although PF6 may have some cellular toxicity due to the chloroquine, in terms of transfection, it was the most preferable carrier among all the derivatives.

For the treatment of dermatologic diseases, Hsu & Mitragotri (2011) had introduced a CPP referred to as SPACE (Skin Penetrating And Cell Entering). This CPP had shown the ability to cross the cell membrane in vivo and in vitro of various skin cells (e.g. keratinocytes, fibroblasts, and endothelial cells) via macropinocytosis. SPACE-conjugated siRNA significantly down-regulated the gene expression of IL-10 (InterLeukin-10), GAPDH (GlycerAldehyde 3-Phosphate DeHydrogenase), and GFP. These CPP-siRNA conjugates were highly proficient medicaments for dermatologic diseases.

In another study, Z. Tai et al. (2015) had introduced four stearylated CPPs (SHRss) synthesized by arginine, histidine, cysteine, and stearyl moieties. Intramolecular cross-linking of these CPPs via disulfide bonds could build polypeptides with different molecular weights (SHRss1, SHRss2, SHRss3, and SHRss4). The CPPs effectively condensed siRNA into stable nanocomplexes. The stability of the SHRss-siRNA nanocomplexes was examined in 50% FBS, and the results revealed that these CPPs can protect the siRNA from complete degradation for up to 48 h, which is nearly double of the parent (without stearyl moiety) CPP. The resulting nanocomplexes (MW∼15 kDa) have shown high cellular uptake activity and rapid dissociation of the siRNA in the cytosol. Interestingly, the release siRNA from the nanocomplexes was still observed in the cytoplasm even after 24 h incubation. Also, the *in vivo* studies successfully showed that SHRss-siRNA nanocomplexes possessed high cellular uptake and gene silencing efficiency.

Triple-negative breast cancer (TNBC) is an aggressive cancer subtype defined by the absence of various receptors such as estrogen, progesterone and human epidermal growth factor receptor 2. The absence of these receptors was a major challenge for TNBC treatment. Recently, S. Galdiero et al. introduced a membranotropic CPP entitled as gH625, which was able to translocate siRNA into triple negative breast cancer cells (Ben Djemaa et al., 2018). A hybrid nanovector was synthesized by fluorescent SPIONs coated with PEG polymer, chitosan polymer, poly-L-arginine, and the CPP. Each component had a specific function: SPION played as a contrast agent and scaffold; cyanine 5.5 for fluorescent labeling; PEG for colloidal stability and immune stealthiness; gH625 for enhancing internalization of the carrier into cells; chitosan and poly-L-arginine (PLR) for siRNA complex formation and favoring the endosomal escape. Thereby, authors developed a magnetic nanocarrier called CPP-capped stealth magnetic nanovectors (CS-MSN) which have shown high serum-protection and siRNA retention even after incubation for 4 hr in a quite high serum concentration (e.g. 50%). For cytotoxicity assessments, different concentrations of CS-MSN were treated to MDA-MB231 cells with different time periods. After incubation at 100 nM concentration for 48 hr, cell viability remained more than 80%, indicating CS-MSN have a limited cell toxicity. The overall results suggest that CS-MSN is a proficient and safe nanocarrier for siRNA delivery.

I. Ullah et al. (2017) introduced a modified CPP, RVG-9R3LC, for improved endosomal escape and transfection of siRNA into cells. This modified CPP enhanced the target gene silencing by >20%, compared to unmodified CPP. When a CPP such as nona-arginine (9R) was attached to a cell receptor-binding ligand like Rabies virus glycoprotein (RVG), the resulting conjugate delivered siRNA effectively by ligand-induced receptor endocytosis. However, it was found that high amount of siRNA complexes remained trapped in endosomes. To improve the endosomal escape, 3 leucine (Leu) and one cysteine residues were incorporated into the CPP sequence to produce RVG-9R3LC. Addition of more than 3 Leu residues showed high cell toxicity, particularly at high CPP concentration. Consequently, it was suggested that RVG9R3LC is nontoxic and safe to cells, and enable endosomal membrane disruption for improved siRNA delivery into the cytoplasm.

Y. W. Lee at al. (2018) investigated the efficacy of cancer-specific CPP, BR2, with anti-vascular endothelial growth factor siRNA (siVEGF). VEGF is normally overexpressed in many cancer cells and closely responsible tumor growth process, angiogenesis. The BR2-siRNA formed stable nanocomplexes with size <200 nm at CR <8, which are suitable for efficient cellular uptake and biodistribution. The serum stability was investigated in 50% FBS at 37 °C for 4 h and the results have shown that BR2 is able to protect siRNA in serum against degradation. In comparison of the transfection degree between normal and cancer cells, it was found that BR2-siVEGF was ∼25% more likely to enter cancer cells than normal cells, which means the nanocomplexes have more propensity and selectivity toward cancer cells.

Recently, S. Hyun et al., (2018) reported a histidine-containing hydrocarbon stapled CPP, LKH-stEK, which has shown the capabilities for efficient siRNA delivery *in vitro* and *in vivo*. It was reported that the incorporation of histidine residues can promote endosomal escape by disrupting the endosomal membrane via proton abstraction mechanism inside endosomes. The LKH-stEK nanocomplexes have shown >90% *in vitro*, and about 50–60% *in vivo* gene knockdown efficiency. It was suggested that this is the first experience of His-containing hydrocarbon stapled CPP for siRNA delivery both *in vitro* and *in vivo*.

## Conclusions

Since the discovery of CPPs and their ability to cross the cell membrane, they have been used for delivery of diverse biomolecules. Many CPP-mediated methods have been introduced due to the requirement for development of safe and effective carriers for drug delivery. In this review, we described how CPPs are biologically safe, have great promise, and are paving the way for improved drug delivery. In particular, some recent siRNA delivery approaches were summarized here.

From previous studies, it has been demonstrated that conjugation of siRNA with most of the unmodified CPPs could not attain a desirable effect due to either lack of serum stability, endosomal entrapment, or some other factor. To overcome these obstacles, chemical modifications of the CPPs and/or siRNAs were performed, which enhanced the transfection efficiency. Compared to conventional methods, CPP-mediated delivery methods have more compelling advantages in terms of efficiency, siRNA-carrying capacity, and biocompatibility. However, an understanding of the CPP internalization pathway is complicated and not fully understood. Therefore, dissecting the mechanism of CPP cellular uptake will help to develop other novel CPPs with enhanced delivery and cell penetration capabilities. The arrival of CPPs is providing new opportunities for systemic siRNA delivery.
